# Major Odorants Released as Urinary Volatiles by Urinary Incontinent Patients

**DOI:** 10.3390/s130708523

**Published:** 2013-07-03

**Authors:** Sudhir Kumar Pandey, Ki-Hyun Kim, Si On Choi, In Young Sa, Soo Yeon Oh

**Affiliations:** 1 Atmospheric Environment Laboratory, Department of Environment & Energy, Sejong University, Seoul 143-747, Korea; E-Mail: skpbhu@gmail.com; 2 Kimberly-Clark Corporation 81, Digital Valley-ro, SuJi-gu, YongIn-si, GyeongGi-do 448-160, Korea; E-Mails: sion.choi@kcc.com (S.C.); inyoung.sa@kcc.com (Y.S.); sooyeon.oh@kcc.com (S.Y.)

**Keywords:** incontinence, urinary, volatiles, GC, HPLC, incontinence pads, sample collection, glass impinger

## Abstract

In this study, volatile urinary components were collected using three different types of samples from patients suffering from urinary incontinence (UI): (1) urine (A); (2) urine + non-used pad (B); and (3) urine + used pad (C). In addition, urine + non-used pad (D) samples from non-patients were also collected as a reference. The collection of urinary volatiles was conducted with the aid of a glass impinger-based mini-chamber method. Each of the four sample types (A through D) was placed in a glass impinger and incubated for 4 hours at 37 °C. Ultra pure air was then passed through the chamber, and volatile urine gas components were collected into Tedlar bags at the other end. These bag samples were then analyzed for a wide range of VOCs and major offensive odorants (e.g., reduced sulfur compounds (RSCs), carbonyls, trimethylamine (TMA), ammonia, *etc.*). Among the various odorants, sulfur compounds (methanethiol and hydrogen sulfide) and aldehydes (acetaldehyde, butylaldehyde, and isovaleraldehyde) were detected above odor threshold and predicted to contribute most effectively to odor intensity of urine incontinence.

## 1. Introduction

Urinary incontinence (UI) is any involuntary leakage of urine which has a profound impact on quality of life [1]. UI is a common condition among women, especially in the elderly [2]. Approximately 50% of all incontinent women are classified as stress incontinent; however, this percentage can be lower in old age [3]. As the prevalence of urge incontinence seems to increase with advancing age, motor urge incontinence (detrusor instability) is often claimed as the most common type of UI in the elderly [4]. Although there are certain medical devices and treatments available for different incontinence types, patients do not often seek for professional help due to shame and embarrassment [5]. To avoid such situation, incontinence pads (*i.e.*, small impermeable multi-layered sheet with high absorbency) are common in practice to absorb unregulated release of urine. The main benefit of using these absorbent incontinence pads is preventing leakage, but there are other key characteristics that absorbent product users look for in day time use. Primary needs from these patients include: “to hold urine, to contain smell, to stay in place, discreetness, and comfort when wet” [6]. Among these needs, odor control is one area that still requires a lot of basic research.

Although analysis of liquid phase urine samples has been a very common practice in clinical diagnostics, gas phase volatile odorants in urine samples have scarcely been evaluated. Zlatkis *et al.* [7] studied volatile metabolites present in human urine and identified 2-butanone, 2-pentanone, 4-heptanone, dimethyl disulfide (DMDS), alkylfurans, pyrrole, and carvone as the characteristic volatile constituents in normal urine. These authors also found some compounds at very high level (*i.e.*, pyrazines, cyclohexanone, lower aliphatic alcohols, and octanols) in urine samples of diabetes mellitus patients under insulin treatment. In a similar effort, a number of low-molecular-weight metabolites (e.g., aliphatic alcohols like ethanol, *n*-propanol, isobutanol, *n*-butanol, and isopentanol, and ketones like 4-heptanone and cyclohexanone) were suggested as marker components of metabolic disorders related to diabetes mellitus because of their presence at abnormally increased concentrations [8]. Urinary volatiles have also been utilized to diagnose metabolic disorders such as phenylketonuria (PKU), maple syrup urine disease (MSUD), isovaleric acidemia, or trimethylaminuria (fish-odor syndrome) [9]. Zhang *et al.* [10] suggested the possibility that routine quantification of volatile methylamines and stable trimethylamine (TMA) N-oxide present in human urine can be used as a diagnostic tool for fish-odor syndrome. In continuation to this effort, Mills *et al.* [11] were also able to determine TMA from urine samples of patients suffering from fish-odor syndrome. Moreover, Wahl *et al.* [12] were able to detect 34 VOCs including TMA and 4-heptanone as metabolites of diagnostic relevance.

Despite some studies conducted on urinary volatiles, they do not fully explain what contributes to urine malodor around patients with urine incontinence. For incontinence pad users, the odor they have to face is not only urine itself, but a used pad could have urine, skin secretions, and vaginal secretions that could interact with skin bacteria in the pad. Used absorbent pads from incontinent patients were tested for gas volatiles in a study done by Norberg *et al.* [13], but only ammonia was studied since ammonia has been long thought to be a main cause of the foul smell of urine around patients. It would be valuable to identify characteristic volatiles from the used pads which could lead to the best odor control solution. Nonetheless, relatively little information is as yet available to characterize urinary volatiles for these cases. In addition, it is also difficult to know at what level these volatiles exist from previous studies which are essential to identify volatiles that exist above the human nose threshold [14].

In this study, urinary volatiles of UI patients were collected from three different types of urinary matrices (urine itself, urine on pad, and used pad) using a unique glass impinger-based chamber system. The urine gas samples collected from different sample types were then analyzed for a wide range of volatiles including a list of 22 offensive odorants designated by the Korean Ministry of Environment (KMOE) [15]. These include two nitrogenous compounds (*i.e.*, ammonia and trimethylamine), four reduced sulfur compounds (CH_3_SH, H_2_S, DMS, and DMDS), five aldehydes (acetaldehyde, propionaldehyde, butyraldehyde, valeraldehyde, and isovaleraldehyde), three aromatic VOCs (styrene, toluene, and xylene), two ketones (MEK and MIBK), on alcohol (i-BuOH), one ester (BuOAc), and four organic acids (propionic, butyric, isovaleric, and valeric acid). The present study focused on the analysis of these offensive odorants from the urine samples between different treatments. In addition, GC-MS combined with a TD system was also employed to obtain data sets for a wide profile of VOCs.

## 2. Materials and Methods

### 2.1. Urine Samples and Treatments

This study was conducted according to the Declaration of Helsinki on Biomedical Research Involving Human Subjects (Somerset West Amendment; World Medical Association, 1996) and was approved by the Ethics Committee of Kimberly Clark. The collection of urinary volatiles was made using urine samples collected in three different manners: (1) urine sample (A); (2) urine artificially applied on a fresh pad (B); and (3) the pads worn by UI patients containing urine excretions (C) (refer to Table 1 for details). The patients were suffering from urinary incontinence. The number of patients was restricted due to unavailability of patients at the hospital of the medical team with which we collaborated to obtain samples. Basically two types of urine samples, *i.e.*, A and C were received from three different patients as described in Table 1. However, the sample type B (urine on pad) was prepared in the laboratory using fresh incontinence pads and applying 30 mL of urine (A) on it. In addition, samples from non-patient, *i.e.*, a urine sample applied on a fresh pad (prepared and named as D) were also collected and analyzed as reference data set. The gaseous samples were basically collected immediately using the collection system to avoid any alteration in chemical composition. Prior to the collection of urinary volatiles, all the raw samples received were stored in a refrigerator at a temperature of 5 °C.

### 2.2. The Scheme and Set up for the Collection of Urinary Volatiles

**Figure 1.**
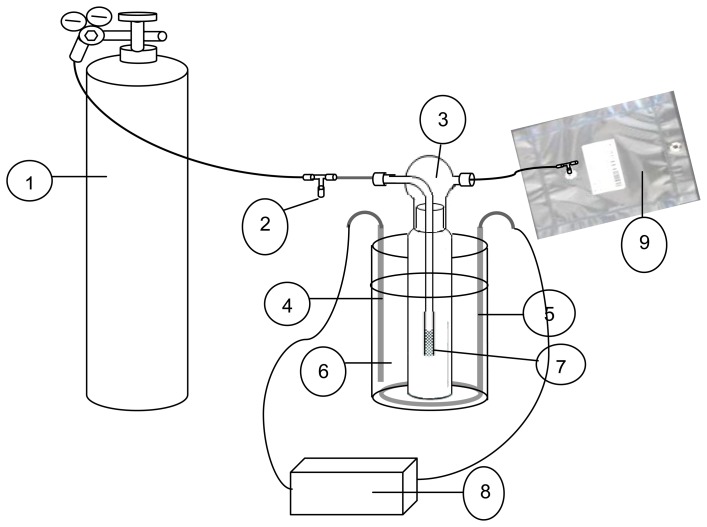
Schematic for sampling procedure applied to collect urinary volatiles from incontinence patients. 1. ultra pure air; 2. flow control; 3. glass impinge; 4. sensor (temperature); 5. heater; 6. samples of (A) to (D) types; 7. bubbler; 8. temperature regulator; 9. tedlar bag to collect volatiles.

In this study, the collection of urinary volatiles was made with a unique glass impinger system (Figure 1). The collection of volatiles was conducted by passing the ultra pure air through the impinger into a Tedlar bag. As shown in Figure 1, all the urine samples obtained in a different manner (A through D) were finally placed in a 500 mL capacity glass impinger. The glass impinger containing each type of urine sample was then placed in a water bath at a controlled temperature of 37 °C (body temperature). These samples were initially incubated for 4 h at normal body temperature (37 °C). This time was selected as incubation time for being representative of the period between pad changes of a typical incontinent patient. After the incubation period, ultrapure air was brought into the camber and passed through the impinger of which outlet is connected to the Tedlar bag. The flow rate was controlled through flow regulators and maintained at 100 mL·min^−1^. As the collection capacity of Tedlar bag was 10 L, the sampling was performed for 100 min. In order to collect the four different types of urine samples (A through D) simultaneously, we used a laboratory build flow split system in which ultrapure air is continuously flushed through the chamber at the above supply rate. The collected gaseous samples were immediately handed over to the respective operator for their quantification.

### 2.3. The Analysis of Urinary Volatiles and Dilution-to-Threshold (D/T) Ratios in Gaseous Phase

In order to quantify a wide profile of urinary volatiles and offensive odorants emitted from urinary samples, various instrumental setups were employed that included universal detection systems such as GC-MS combined with a thermal desorber (TD). In addition, specific analytical techniques for the target odorants are briefly summarized in Table 2(b). The GC-MS system was first calibrated with gaseous standard of seven VOC mixtures (benzene, toluene, xylene, MEK, i-BuOH, MIBK, and BuOAc). Note that all these compounds (except benzene) belong to a list of offensive odorants managed by the KMOE [15]. All other VOCs were then identified through library search and spectral interpretations and quantified by carbon number ratio technique [16,17]. The details on operating conditions used to quantify the selected target odorants and the related QA/QC conditions are comparable to those reported in our recent study [18].

In addition to quantitation of individual odorants, actual application of olfactometry was also made in terms of dilution-to-threshold (D/T) ratio by the air dilution sensory (ADS) test which is the standard procedure established by the KMOE [15,19]. The KMOE ADS test method sets a threshold in which the central trend of odor index value is derived geometrically for a given odor sample, after excluding the data sets of extreme cases. Being developed and modified from the triangle odor bag method of Japan [20], it is currently the main test method in Korea. Samples were analyzed by a panel of five members; all of these members were selected based on a pre-screening test in which all participants are requested to distinguish samples of deionized water from testing solutions containing four chemicals with the following weight (%) values: acetic acid (1), TMA (0.1), methylcyclo-pentenolone (3.2), and β-penylethyl alcohol (1). Details of the ADS test are described in Kim *et al.* [19].

## 3. Results and Discussion

In the Supplementary Information (SI), a statistical summary is presented in Tables S1 and S2 to describe the results (both offensive odorants and VOC, respectively) of three replicates for different sample treatment types: A, B, C, (for three patients) and D (for a pooled sample of three non-patients). In order to measure sensory perception of each treatment sample, the dilution-to-threshold (D/T) ratio was examined for each treatment by five panel members (see Table 3).

For this test, odor panels conducted sensory evaluation of the worn pad sample, which might best represent the actual situation of pad-wearing incontinence patients. Worn pad samples were measured as the most odorous one among three different matrices. However, there was not any significant difference in D/T values across different treatments, when the t-test was applied, as seen from relatively large variabilities (e.g., high standard deviation).

**Figure 2.**
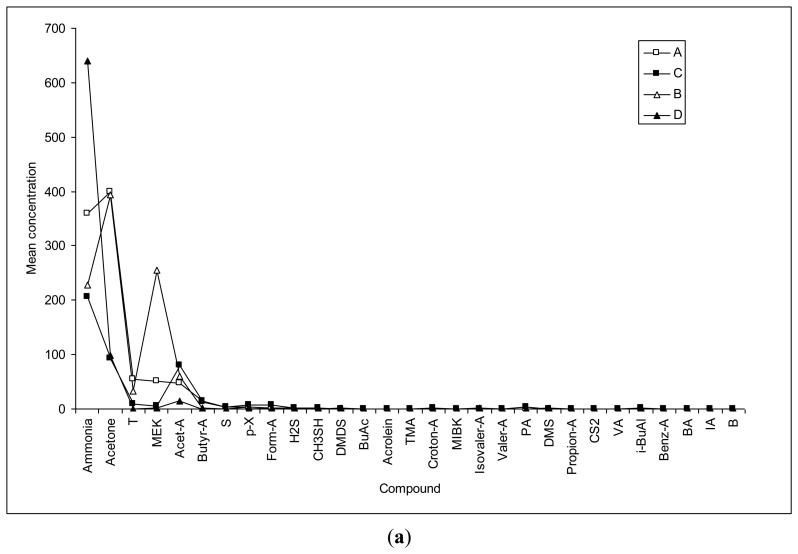

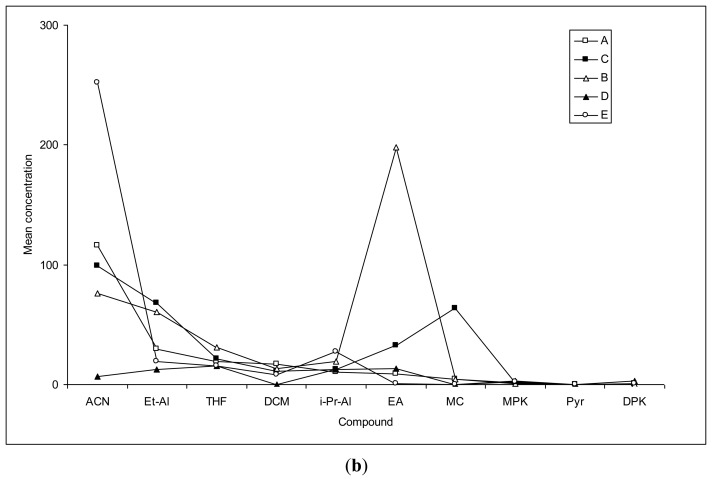
Comparison of the mean concentration (ppb) of urinary volatiles between samples of different treatments (ACN = acetonitrile, Et-Al = ethyl alcohol, THF = tetrahydrofuran, DCM = dichloromethane, i-Pr-Al = isopropyl alcohol, EA = ethyl acetate, MC = methylene chloride, MPK = methyl propyl ketone, Pyr = pyrrole, and DPK = dipropylketone): (**a**) Offensive odorants listed by KMOE (acetone, acrolein, croton-A. CS_2_, Benz-A, and benzene are not in the list but are shown with their respective groups); and (**b**) All dominant VOCs determined with GC-MS in addition to the abovementioned offensive odorants.

To investigate the compounds that contribute to the odor around incontinent patients, volatiles which exceed the odor threshold level were screened. Although concentrations of each volatile in urine samples can vary, it was assumed that odorants exceeding the odor threshold level contribute the most to the odor around the incontinence pad users. As shown in Figure 2, the chemicals detected from both patients and non-patients are compared in terms of the magnitude of the observed concentration data. In this respect, relative dominance of the odorant species is distinguished in Figure 2, as comparison is made for both official offensive odorants and most VOCs detected by GC/MS. However, the results compiled in concentration data alone are not fully meaningful. Hence, to overcome such limitation, the actual contribution of odorant species is determined by the relationship between their concentration and threshold which is commonly represented as odor activity values (OAV).

To learn more about this delicate relationship between odorant species, our target chemicals that were found to exceed the odor threshold values in this study are sorted out and compared in Table 4. If the results are evaluated simply in terms of frequency of such exceedance between four sample types, the contribution of five compounds seems to be most evident among all measured species: their relative ordering is found on the order of acetaldehyde (4), butyraldehyde (4), methanethiol (3), hydrogen sulfide (2), and isovaleraldehyde (1). Sulfur and aldehyde compounds have long known to be one of the significant volatiles from various biological matrices. Because of their low thresholds, it is possible that these volatiles should contribute to the foul smell of urine [21]. Considering incontinent people have to wear pads instead of flushing urine directly into a toilet (like non-patients), patients would more readily be able to detect these odors.

Although the concentration of most detected volatile components were below their threshold, it is possible that the sensory detection of their mixture can be made more easily than that of individuals. Previous studies showed that subjects can often detect each of two odors (from their mixture) at half of their individual threshold concentrations [22]. Due to the limited number of sample, it is difficult to distinguish which of those sub-threshold components may exert more influences on the overall odor. However, in terms of the detection frequency of each compound, propionic acid, isobutyl alcohol, pentamethylene, ethyl acetate, nonane, and *o*-xylene could be potential candidates in this respect. This is because their abundance in worn pad samples (C) was higher by about two times or more than that of patients' fresh urine (A) (see Table 5). Comparison of the detection frequency between urine-insulted new pad (B) and worn pad (C) showed lower abundance of propionic acid and pentamethylene in the former, while the others have same abundance between them. Therefore, one can guess that propionic acid and pentamethylene would come from bacteria interaction or vaginal secretion, while others were from pad or interaction between urine and pad.

## 4. Conclusions

In this study, a unique glass impinger-based mini-chamber method was introduced for collection of urinary volatiles from three different types of volatile samples of incontinent patients. The proposed method was simple, easy to operate, and reliable enough to yield a wide range of VOCs that can be suitably analyzed with common available analytical methods. It was demonstrated from this study that used incontinence pads with urine excretion had high sensory dilution/threshold ratio, and the resulting volatiles could include sulfur compounds (methanethiol and hydrogen sulfide) and aldehydes (acetaldehyde, butylaldehyde, and isovaleraldehyde).

Ammonia levels were low in our study. For incontinent patient samples, none showed concentrations above the human nose threshold (1,500 ppb). Ammonia was long considered to be main cause of urine odor from urea by bacterial urease. As ammonia is produced from bacterial enzymatic reaction, its production might be a delayed action which is reason of low concentration in this study condition which involved a 4 hour incubation at body temperature.

Causative VOCs for higher sensory dilution/threshold ratio of a used pad (B) still need to be investigated. Some VOCs that were higher in used pads then in urine alone include propionic acid, butylaldehyde, formaldehyde, pentamethylene, ethyl acetate, and *o*-xylene. Propionic acid is one of the acids that are detected in vaginal secretions for acid-producing women. Work from Huggins *et al*. [23] showed two distinct types of females with respect to production of the C_2_–C_5_ aliphatic acids, and propionic acid was one of these acids. It is presumed that an increase amount of propionic acid in used pads *versus* artificially insulted pad could be due to vaginal secretions from the users. However, none of the matrices exceeded the threshold level for propionic acid and other VOCs. It could be possible to explain that even small quantities of various odorants could contribute to overall odor by “synergy impact of mixture”.

Interpersonal variability was high in this study just as other biological samples. It would require bigger sample size to reach better conclusions about the result. Despite the small sample size and high interpersonal variability, the research reported here could provide a valuable basis to study volatiles from incontinent patients with new quantification method and matrices used (urine, insulted in pad, and used pad). Further studies are required to reconfirm this result and to investigate effects of diet, race, age, disease, and pad type. It is anticipated that our research could lead to interpretation of the characteristics of urine volatiles from different matrices.

## Figures and Tables

**Table 1.** Basic experimental scheme for the analysis of urinary volatiles collected from female incontinence patients and normal subjects.

(**a**) Sample treatment				
A a		30 mL of patient's urine in an impinger		
B b		30 mL of patient's urine on fresh incontinence pad		
C c		pad worn by incontinent patients		
D b		30 mL of a non-patient's urine on a fresh incontinence pad		
(**b**) Description of each experiment.				

**Order**	**Experiment**	**Description**	**Age (Years)**	**Samples Collected**

1	E1	Patient-1	58	A1, B1 and C1
2	E2	Pateint-2	49	A2, B2 and C2
3	E3	Patient-3	62	A3, B3 and C3
4	E4	Pool of 4 persons	54–72	D1
5	E5	Non-patient person-2	50	D2
6	E6	Non-patient person-3	74	D3

a Ultra pure air was passed through the urine placed in a glass impinger from one end, and the volatiles were collected in a 10 L Tedlar bag connected at the other end;

b Urine was applied on fresh incontinence pad, and sampling was made with the same procedure described above;

c The incontinence pad worn by the patients were placed in the glass impinger, and volatiles were collected in Tedlar bag with the same procedure described above.

**Table 2.** Sample preparation and procedures for collecting the urinary volatiles in a 10 L Tedlar bag.

(**a**) Sampling condition	
Size of the impinger	500 mL
Incubation temperature	37 °C
Incubation time	4 hours
Gas used	Ultra pure air
Flow rate	100 mL·min ^−1^
Duration of sampling	100 min.
Total vol. of air passed	20 L
Number of bags collected	2
(**b**) Target analytes and their determination by different analytical systems.	

[1] Direct method	
Dilution/threshold ratio	Sensory evaluation
[2] Indirect (instrumental) method	
(i) Gas chromatography (GC) methods	
Organic acids	Thermal desorption (TD)-gas chromatography (GC)-flame ionization detector (FID)
Trimethylamine	Solid phase microextraction (SPME)-GC-nitrogen phosphorous detector (NPD)
All possible VOCs	TD-GC-mass spectrometer (MS)
Reduced sulfur compounds (RSCs)	TD-GC-pulsed flame photometric detector (PFPD)
(ii) High performance liquid chromatography (HPLC) method	
Carbonyls	
(iii) Others	
Total hydrocarbon (THC)	Micro-FID
Ammonia	Ultraviolet visible (UV-VIS) spectrometer

**Table 3.** Summary of air dilution sensory test for different treatments of urine samples.

	**Dilution-to-Threshold (D/T) Ratio**			

	**Treatment Type A**	**Treatment Type B**	**Treatment Type C**	**Treatment Type D**
Mean ± SD (Median) a	68.8 ± 67.3 (54.8)	106 ± 88.8 (66.9)	243 ± 367 (44.0)	106 ± 64.7 (100)
min-max	9.65-142	44.0-208	25.0-669	44.0-173

(a) Treatment types A, B, and C represents mean of three patients (For instance, A is mean of A1, A2, and A3 as described in Table 1). Treatment type D is the mean of three non-patients' experiment (*i.e.*, D1, D2, and D3 as described in Table 1).

**Table 4.** A list of compounds exceeding odor threshold level in this investigation.

	**Mean Concentration (ppb)**				**Odor Threshold (ppb)**^a^

	**Patient** **Urine**	**Patient New** **Pad**	**Patient Worn** **Pad**	**Normal New** **Pad**	
Hydrogen sulfide	1.37	ND b	0.87	ND	0.41
Methanethiol	0.96	0.15	1.97	ND	0.07
Acetaldehyde	47.21	60.29	79.59	117.80	1.5
Butyraldehyde	14.82	0.93	13.08	2.94	0.67
Isovaleraldehyde	ND	ND	ND	1.5	0.1

a Nagata [20]

b ND: below detection limit.

**Table 5.** Components that had ≥ 2 higher abundance in worn pad (sample C) than urine sample (sample A).

**Sample Type**	**Abundance**			

	**A**	**B**	**C**	**D**
Propionic Acid	1	1	3	2
Isobutyl alcohol	0	2	2	0
Pentamethylene	0	0	2	0
Ethyl acetate	1	3	3	1
Nonane	0	2	2	0
*o*-Xylene	1	3	3	0

## Supplementary Files

Supplementary File 1 Supplementary Information (PDF, 174 KB)
