# Supplementary material for: Major Odorants Released as Urinary Volatiles by Urinary Incontinent Patients
Source: Sensors (Basel). 2013 Jul 3;13(7):8523–33. doi: 10.3390/s130708523 (PMC3758608; doi:10.3390/s130708523)
Supplement: Supplementary File 1 — Supplementary Information (PDF, 174 KB) [file sensors-13-08523-s001.pdf]

OPEN ACCESS

sensors

ISSN 1424-8220

www.mdpi.com/journal/sensors

Supplementary Information

Major Odorants Released as Urinary Volatiles by Urinary Incontinent Patients. *Sensors* 2013, *13*, 8523-8533

Sudhir Kumar Pandey [1,†], Ki-Hyun Kim [1,*], Si On Choi [2], In Young Sa [2] and Soo Yeon Oh [2]

[1] Atmospheric Environment Laboratory, Department of Environment & Energy, Sejong University, Seoul 143-747, Korea; E-Mail: skpbhu@gmail.com

[2] Kimberly-Clark Corporation 81, Digital Valley-ro, SuJi-gu, YongIn-si, GyeongGi-do 448-160, Korea; E-Mails: sion.choi@kcc.com (S.C.); inyoung.sa@kcc.com (Y.S.); sooyeon.oh@kcc.com (S.Y.)

† Current address: Department of Botany, Guru Ghasidas Central University, Bilaspur C.G. 495009, India.

* Author to whom correspondence should be addressed; E-Mail: khkim@sejong.ac.kr; Tel.: +82-2-499-9151; Fax: +82-2-3408-4320.

Table S1. Summary of concentrations of offensive odorants released by different treatments of urine samples from the incontinence patients and normal person.

Group	Full Name	Abbreviations		Concentration (ppb) [a b]				
				Treatment Type A	Treatment Type B	Treatment Type C	Treatment Type D	Treatment Type E
RSC	Hydrogen sulfide	H_2S	Mean ± SD (Median)	1.47 ± 1.17 (1.46)	1.03 ± 1.38 (0.31)	0.22 ± 0.08 (0.17)	1.29	0.50 ± 0.69 (0.10)
			min-max	0.31-2.65	0.17-2.62	0.17-0.31		0.10-1.29
	Methyl mercaptan	CH_3SH	Mean ± SD (Median)	1.11 ± 0.60 (1.28)	2.18 ± 3.25 (0.44)	0.35 ± 0.16 (0.44)	1.28	0.50 ± 0.67 (0.11)
			min-max	0.44-1.61	0.16-5.92	0.17-0.44		0.10-1.28
	Dimethyl sulfide	DMS	Mean ± SD (Median)	0.15 ± 0.04 (0.16)	0.37 ± 0.46 (0.03)	0.13 ± 0.04 (0.11)	1.37	0.58 ± 0.70 (0.33)
			min-max	0.11-0.19	0.10-0.90	0.10-0.17		0.04-1.37
	Carbon disulfide	CS_2	Mean ± SD (Median)	0.13 ± 0.17 (0.03)	0.07 ± 0.06 (0.03)	0.30 ± 0.47 (0.03)	0.60	0.26 ± 0.29 (0.11)
			min-max	0.03-0.32	0.03-0.13	0.03-0.85		0.08-0.60
	Dimethyl disulfide	DMDS	Mean ± SD (Median)	0.69 ± 0.67(0.63)	0.72 ± 1.19 (0.05)	1.23 ± 1.40 (0.03)	0.79	0.53 ± 0.37 (0.68)
			min-max	0.05-1.38	0.02-2.09	0.03-0.85		0.10-0.79
Carbonyls	Formaldehyde	Form-A	Mean ± SD (Median)	1.70 ± 1.54 (1.01)	7.95 ± 5.77 (6.26)	1.84 ± 0.84 (1.46)	2.57	5.40 ± 5.25 (1.46)
			min-max	0.63-3.47	3.22-14.4	1.27-2.81		2.10-11.5
	Acetaldehyde	Acet-A	Mean ± SD (Median)	47.2 ± 64.8 (10.6)	79.6 ± 81.5 (66.2)	60.2 ± 42.6 (24.1)	14.5	118 ± 107 (89.1)
			min-max	9.08-122	5.56-167	11.4-145		28-236
	Acrolein	Acrolein	Mean ± SD (Median)	0.31 ± 0.14 (0.39)	0.31 ± 0.14 (0.39)	0.31 ± 0.14 (0.39)	0.51	0.27 ± 0.21 (0.15)
			min-max	0.15-0.39	0.15-0.39	0.15-0.39		0.15-0.51
	Acetone	Acetone	Mean ± SD (Median)	399 ± 267 (322)	92.7 ± 79.3 (128)	393 ± 267 (313)	98.4	162 ± 112 (100)
			min-max	179-697	1.93-148	175-691		175-691
	Propionaldehyde	Propion-A	Mean ± SD (Median)	0.14 ± 0.01 (0.15)	0.14 ± 0.01 (0.15)	0.14 ± 0.01 (0.15)	0.63	0.63 ± 0.53 (0.58)
			min-max	0.13-0.15	0.13-0.15	0.13-0.15		0.13-0.63
	Crotonaldehyde	Croton-A	Mean ± SD (Median)	0.21 ± 0.15 (0.12)	2.49 ± 3.86 (0.41)	0.13 ± 0.004 (0.12)	0.58	0.64 ± 0.53 (0.58)
			min-max	0.12-0.38	0.12-6.95	0.12-0.13		0.14-1.20
	Butyraldehyde	Butyr-A	Mean ± SD (Median)	14.9 ± 25.6 (0.14)	13.1 ± 18.8 (3.06)	1.02 ± 1.53 (0.14)	0.75	3.19 ± 3.05 (2.22)
			min-max	0.13-44.4	1.40-34.8	0.13-2.78		0.75-6.61

Table S1. *Cont.*

Group	Full Name	Abbreviations		Concentration (ppb) [a b]				
				Treatment Type A	Treatment Type B	Treatment Type C	Treatment Type D	Treatment Type E
	Benzaldehyde	Benz-A	Mean ± SD (Median)	0.12 ± 0.004 (0.16)	0.12 ± 0.004 (0.13)	0.12 ± 0.004 (0.13)	0.83	0.36 ± 0.40 (0.13)
			min-max	0.12-0.13	1.40-34.8	0.12-0.13		0.13-0.83
	Isovaleraldehyde	Isovaler-A	Mean ± SD (Median)	0.17 ± 0.03 (0.16)	0.17 ± 0.03 (0.16)	0.17 ± 0.03 (0.13)	0.97	1.82 ± 1.73 (0.97)
			min-max	0.15-0.21	0.15-0.21	0.12-0.13		0.68-3.82
	Valeraldehyde	Valer-A	Mean ± SD (Median)	0.17 ± 0.02 (0.18)	0.17 ± 0.02 (0.18)	0.17 ± 0.02 (0.18)	0.69	0.35 ± 0.29 (0.18)
			min-max	0.15-0.20	0.15-0.20	0.15-0.20		0.18-0.69
Acid	Propionic acid	PA	Mean ± SD (Median)	0.15 ± 0.24 (0.01)	3.04 ± 4.25 (0.75)	0.29 ± 0.49 (0.01)	0.64	0.33 ± 0.29 (0.44)
			min-max	0.005-0.43	0.42-7.94	0.005-0.86		0.01-0.55
	Butyric acid	BA	Mean ± SD (Median)	0.01 ± 0.005 (0.01)	0.011 ±0.007 (0.009)	0.01 ± 0.002 (0.01)	0.01	0.17 ± 0.28 (0.01)
			min-max	0.005-0.01	0.005-0.018	0.005-0.01		0.01-0.48
	Isovaleric acid	IA	Mean ± SD (Median)	0.01 ± 0.005 (0.01)	0.07 ± 0.12(0.01)	0.01 ± 0.002 (0.01)	0.01	0.21 ± 0.34 (0.01)
			min-max	0.005-0.01	0.005-0.21	0.005-0.01		0.01-0.60
	Valeric acid	VA	Mean ± SD (Median)	0.07 ± 0.10 (0.01)	0.12 ± 0.19 (0.01)	0.07 ± 0.06 (0.10)	0.03	0.01 ± 0.01 (0.01)
			min-max	0.01-0.19	0.01-0.35	0.01-0.08		0.01-0.03
N compounds	Ammonia	NH_3	Mean ± SD (Median)	359 ± 264 (465)	207 ± 256 (58.7)	228 ± 220 (149)	639	1114 ± 614 (1391)
			min-max	59-553	58.5-502	58.5-477		410-1540
	Trimethylamine	TMA	Mean ± SD (Median)	0.18 ± 0.05 (0.17)	0.18 ± 0.05 (0.17)	0.18 ± 0.05 (0.17)	0.31	0.21 ± 0.08 (0.17)
			min-max	0.12-23	0.12-0.23	0.12-0.23		0.17-0.31

Table S2. Summary of concentrations of all the VOCs (on GC-MS system) released by different treatments of urine samples from the incontinence patients and normal person.

**VOCs**		**Concentration (ppb)** [a, b]			
[A] Target VOC		Treatment type A	Treatment type B	Treatment type C	Treatment type D
Benzene	Mean ± SD (Median)	0.01 ± 0.01 (0.02)	084 ± 1.42 (0.02)	0.01 ± 0.01 (0.02)	0.01
	min-max	0.002-0.02	0.02-2.47	0.002-0.02	
Toluene	Mean ± SD (Median)	55.0 ± 88.1 (6.43)	8.50 ± 4.95 (9.89)	33.7 ± 44.8 (13.2)	0.81
	min-max	1.95-157	2.99-12.6	2.80-85.1	
p-Xylene	Mean ± SD (Median)	3.31 ± 3.56 (1.64)	7.21 ± 8.51 (3.23)	3.99 ± 1.97 (3.76)	0.54
	min-max	0.89-7.40	1.42-17	2.16-6.07	
Styrene	Mean ± SD (Median)	3.41 ± 5.89 (0.01)	3.13 ± 4.43 (1.18)	0.01 ± 0.01 (0.01)	0.01
	min-max	0.01-10.2	0.01-8.21	0.001-0.01	
Methyl ethyl ketone	Mean ± SD (Median)	50.4 ± 80.1 (6.56)	6.31 ± 4.25 (4.72)	255 ± 428 (10.6)	1.69
	min-max	1.82-143	3.07-11.1	6.45-749	
Isobutyl alcohol	Mean ± SD (Median)	0.03 ± 0.02 (0.03)	2.64 ± 2.31 (3.43)	2.24 ± 2.68 (1.46)	0.01
	min-max	0.002-0.04	0.03-4.45	0.03-5.23	
Methyl isobutyl ketone	Mean ± SD (Median)	0.20 ± 0.31 (0.02)	0.01 ± 0.002 (0.02)	0.01 ± 0.002 (0.02)	0.01
	min-max	0.02-0.56	0.01-0.02	0.01-0.02	
Butyl acetate	Mean ± SD (Median)	0.45 ± 0.75 (0.01)	0.01 ± 0.001 (0.01)	0.01 ± 0.001 (0.01)	0.01
	min-max	0.01-1.31	0.01-0.01	0.01-0.01	
[B] Other VOCs through MS					
Isopropyl alcohol	Mean ± SD (Median)	10.6 ± 3.92 (10.0)	12.5 ± 21.6 (0.05)	19.2 ± 7.76 (17.1)	12.6
	min-max	7.02-14.8	0.05-37.4	12.8-27.8	
Pentamethylene	Mean ± SD (Median)	0.03 ± 0.0003 (0.03)	4.89 ± 6.28 (2.63)	0.03 ± 0.0003 (0.03)	0.03
	min-max	0.03-0.03	0.05-12.0	0.03-0.03	
Methylene Chloride	Mean ± SD (Median)	4.45 ± 7.69 (0.01)	63.7 ± 110 (0.01)	4.64 ± 8.03 (0.01)	0.01
	min-max	0.01-13.3	0.01-191	0.01-13.9	
Acetic acid	Mean ± SD (Median)	2.78 ± 4.81 (0.01)	0.01 ± 0 (0.01)	1.75 ±3.02 (0.01)	0.01
	min-max	0.01-8.34	0.01-0.02	0.01-5.24	
Trichloroethylene	Mean ± SD (Median)	0.01 ± 0 (0.01)	15.8 ± 27.4 (0.01)	0.52 ± 0.88 (0.01)	0.01
	min-max	0.01-0.01	0.01-47.5	0.01-1.54	

Table S2. *Cont.*

VOCs		Concentration (ppb) [a, b]			
Acetonitrile	Mean ± SD (Median)	116 ± 48.1 (88.6)	99.0 ± 46.2 (61.1)	75.8 ± 12.9 (74.6)	6.60
	min-max	49.6-209	44.8-191	54.0-98.7	
Ethyl alcohol	Mean ± SD (Median)	29.5 ± 19.2 (35.8)	68.0 ± 59.3 (47.4)	60.6 ±52.0 (50.1)	12.9
	min-max	7.88-44.7	21.7-135	14.6-117	
Dichloromethane	Mean ± SD (Median)	16.7 ± 12.0(15.5)	11.1 ± 19.1 (0.11)	13.5 ± 13.9 (12.6)	0.01
	min-max	5.42-29.3	0.01-33.2	0.01-27.8	
Ethyl acetate	Mean ± SD (Median)	9.19 ± 15.9 (0.03)	32.3 ± 51.5 (5.06)	198 ± 337 (6.15)	13.2
	min-max	0.03-27.5	0.13-91.8	0.53-588	
Butyl alcohol	Mean ± SD (Median)	0.61 ± 1.06 (0.002)	0.002 ± 0 (0.002)	0.002 ± 0 (0.002)	0.002
	min-max	0.002-1.84	0.002-0.002	0.002-0.002	
1-Methoxy-2-propanol	Mean ± SD (Median)	1.39 ± 2.40 (0.002)	0.002 ± 0 (0.002)	1.41 ± 2.45 (0.002)	0.002
	min-max	0.002-4.16	0.002-0.002	0.002-4.24	
Tetrahydrofuran	Mean ± SD (Median)	19.2 ± 5.75 (16.01)	21.1 ± 7.05 (23.0)	30.9 ± 14.4 (27.4)	15.2
	min-max	15.8-25.8	13.3-27	18.6-46.7	
Methyl propyl ketone	Mean ± SD (Median)	1.28 ± 1.54 (0.85)	1.27 ± 2.21 (0.001)	1.09 ± 1.30 (0.73)	2.79
	min-max	0.001-3.00	0.001-3.82	0.001-2.53	
Methyl isopropyl ketone	Mean ± SD (Median)	0.02 ± 0 (0.02)	0.02 ± 0 (0.02)	0.10 ± 0.14 (0.02)	0.02
	min-max	0.02-0.02	0.02-0.02	0.02-0.26	
3,3-Dimethyloxetane	Mean ± SD (Median)	0.99 ± 1.71 (0.001)	0.001 ± 0 (0.001)	0.001 ± 0 (0.001)	0.001
	min-max	0.001-2.97	0.001-0.001	0.001-0.001	
n-Hexane	Mean ± SD (Median)	0.48 ± 0.83 (0.001)	0.001 ± 0 (0.001)	0.31 ± 0.53 (0.001)	0.001
	min-max	0.001-1.44	0.001-0.001	0.001-0.93	
1-Methoxy-2-acetoxypropane	Mean ± SD (Median)	5.30 ± 9.18 (0.001)	0.001 ± 0 (0.001)	0.001 ± 0 (0.001)	0.001
	min-max	0.001-0.14	0.001-0.001	0.001-0.001	
Isothiocyanatocyclohexane	Mean ± SD (Median)	0.05 ± 0.08 (0.001)	0.001 ± 0 (0.001)	0.001 ± 0 (0.001)	0.001
	min-max	0.001-0.14	0.001-0.001	0.001-0.001	
2-Methyl-3-hexanone	Mean ± SD (Median)	0.001 ± 0 (0.001)	0.001 ± 0 (0.001)	0.52 ± 0.90 (0.001)	0.001
	min-max	0.001-0.001	0.001-0.001	0.001-1.55	
Ethylbenzene	Mean ± SD (Median)	0.23 ± 0.31 (0.09)	3.01 ± 4.60 (0.73)	0.74 ± 0.94 (0.41)	0.01
	min-max	0.01-0.59	0.01-8.31	0.01-1.80	

Table S2. *Cont.*

VOCs		Concentration (ppb) [a, b]			
Ethylhexanol	Mean ± SD (Median)	0.001 ± 0 (0.001)	0.39 ± 0.67 (0.001)	0.001 ± 0 (0.001)	0.001
	min-max	0.001-0.001	0.001-1.16	0.001-0.001	
o-Ethyltoluene	Mean ± SD (Median)	0.17 ± 0.29 (0.001)	0.001 ± 0 (0.001)	0.001 ± 0 (0.001)	0.001
	min-max	0.001-0.51	0.001-0.001	0.001-0.001	
2,2,3-Trimethylhexan	Mean ± SD (Median)	0.001 ± 0 (0.001)	0.91 ± 1.56 (0.01)	0.001 ± 0 (0.001)	0.01
	min-max	0.001-0.001	0.01-2.70	0.001-0.001	
Heptane, 2,2,4,6,6-pentamethyl-	Mean ± SD (Median)	0.01 ± 0 (0.01)	2.20 ± 3.80 (0.01)	0.01 ± 0 (0.01)	0.01
	min-max	0.01-0.01	0.01-6.58	0.01-0.01	
l-Limonene	Mean ± SD (Median)	0.01 ± 0 (0.01)	0.008 ± 0.001 (0.008)	0.16 ± 0.27 (0.01)	0.01
	min-max	0.01-0.01	0.008-0.009	0.01-0.47	
n-Nonyl alcohol	Mean ± SD (Median)	0.09 ± 0.15 (0.001)	0.0008 ± 0 (0.0008)	0.001 ± 0 (0.001)	0.001
	min-max	0.001-0.25	0.0008-0.0008	0.001-0.001	
Limonene	Mean ± SD (Median)	0.07 ± 0.12 (0.001)	0.0008 ± 0 (0.0008)	0.15 ± 0.25 (0.001)	0.001
	min-max	0.001-0.21	0.0008-0.0008	0.001-0.44	
Ethylic acid	Mean ± SD (Median)	0.004 ± 0 (0.004)	0.004 ± 0 (0.004)	1.49 ± 2.58 (0.004)	0.004
	min-max	0.004-0.004	0.004-0.004	0.004-4.47	
Methyl pyruvate	Mean ± SD (Median)	0.43 ± 0.74 (0.001)	0.001 ± 0 (0.001)	0.001 ± 0 (0.001)	0.001
	min-max	0.001-1.28	0.001-0.001	0.001-0.001	
Methyl methacrylate	Mean ± SD (Median)	0.001 ± 0 (0.001)	0.50 ± 0.87 (0.001)	2.00 ± 3.46 (0.001)	0.001
	min-max	0.001-0.001	0.001-1.50	0.001-5.99	
2-Ethylhexanol	Mean ± SD (Median)	018 ± 0.31 (0.001)	0.001 ± 0 (0.001)	0.001 ± 0 (0.001)	0.001
	min-max	0.001-0.54	0.001-0.001	0.001-0.001	
Nonane	Mean ± SD (Median)	0.01 ± 0 (0.01)	0.20 ± 0.18 (0.22)	0.19 ± 0.17 (0.22)	0.20
	min-max	0.01-0.01	0.01-0.36	0.01-0.34	
Pyrrole	Mean ± SD (Median)	0.34 ± 0.53 (0.03)	0.03 ± 0 (0.03)	0.30 ± 0.46 (0.03)	0.03
	min-max	0.03-0.95	0.03-0.03	0.03-0.83	
o-X	Mean ± SD (Median)	0.07 ± 0.06 (0.03)	1.38 ± 0.98 (1.40)	1.47 ± 1.11 (0.98)	1.25
	min-max	0.03-0.14	0.39-2.36	0.69-2.73	

Table S2. *Cont.*

VOCs	Concentration (ppb) [a, b]				
3,7-Dimethylnonane	Mean ± SD (Median)	0.01 ± 0 (0.01)	4.80 ± 8.31(0.01)	0.01 ± 0 (0.01)	0.01
	min-max	0.01-0.01	0.01-14.4	0.01-0.01	
2,2,4,6,6-Pentamethylheptane	Mean ± SD (Median)	0.01 ± 0 (0.01)	1.37 ± 2.36 (0.01)	0.01 ± 0 (0.01)	0.01
	min-max	0.01-0.01	0.01-4.10	0.01-0.01	
5-Ethyl-2,2,3-trimethylheptane	Mean ± SD (Median)	0.30 ± 0.51 (0.004)	12.7 ± 13.4 (11.4)	0.001 ± 0 (0.001)	0.001
	min-max	0.004-0.88	0.004-26.8	0.001-0.001	
2,3,5,8-Tetramethyldecane	Mean ± SD (Median)	0.002 ± 0 (0.002)	0.60 ± 1.03 (0.002)	0.002 ± 0 (0.002)	0.002
	min-max	0.002-0.002	0.002-1.79	0.002-0.002	
Dipropyl ketone	Mean ± SD (Median)	0.29 ± 0.44 (0.06)	0.49 ± 0.83 (0.01)	0.02 ± 0.01 (0.01)	2.66
	min-max	0.01-0.80	0.01-1.45	0.01-0.03	
1,3.5 TMB	Mean ± SD (Median)	0.01 ± 0 (0.01)	0.01 ± 0 (0.01)	0.36 ± 0.62 (0.001)	0.40
	min-max	0.01-0.01	0.01-0.01	0.001-0.02	

[a] Treatment types A, B, and C represents mean of three patients (For instance, A is mean of A1, A2, and A3 as described in Table 1). Treatment type E is the mean of three normal person's experiment (*i.e.*, E1, E2, and E3 as described in Table 1); [b] For all the cases of below detection limit, 1/2 of the respective detection limit (DL) were considered for calculation of statistics.

© 2013 by the authors; licensee MDPI, Basel, Switzerland. This article is an open access article distributed under the terms and conditions of the Creative Commons Attribution license (http://creativecommons.org/licenses/by/3.0/).
